# Co-existing Bilateral Pulmonary Embolism and Intra-cardiac Mass: A Case of Catastrophic Antiphospholipid Syndrome-like Disease

**DOI:** 10.7759/cureus.3438

**Published:** 2018-10-10

**Authors:** Huma Zahid, Saira Hassan, Salma Gul, Komal Rizwan, Saif Ullah Khan, Maaz A Maghazi

**Affiliations:** 1 Hematology / Oncology, Shifa International Hospital, Islamabad, PAK; 2 Hematology / Oncology, Shifa International Hospital, Islamabad , PAK; 3 Radiology, Shifa International Hospital, Islamabad, PAK; 4 Internal Medicine, Shifa International Hospital, Islamabad, PAK

**Keywords:** anti phospholipid syndrome, bilateral pulmonary embolism, catastrophic apls, intra cardiac mass, pulmonary embolism

## Abstract

Young patients presenting with thrombotic events like pulmonary embolism and cardiological phenomenon such as presence of an intracardiac mass, without any underlying risk factors, should be promptly investigated for thrombophilias including antiphospholipid syndrome (APLS). This case is reported to highlight rare occurrence of co-existing bilateral extensive pulmonary embolism and an intra-cardiac mass at presentation of antiphospholipid syndrome as well as progression to near catastrophic APLS.

## Introduction

Antiphospholipid syndrome (APLS) is a heterogeneous autoimmune disorder of hyper coagulation. It is manifested mostly as arterial and venous thrombosis, recurrent fetal loss and thrombocytopenia [1] and by presence of antiphospholipid antibodies (aPL) such as anticardiolipin antibodies and lupus anticoagulant [2]. Antiphospholipid syndrome has varying presentations and can manifest in any organ system thus requiring multidisciplinary team for optimal management. The cardiac manifestations include coronary thrombosis, myocardial infarction, cardiomyopathy, valvular involvement and vegetations which may cause systemic and pulmonary emboli [3]. Recurrent thromboembolic events lead to development of catastrophic antiphospholipid syndrome (CAPS)-like disease.

Here we present a case of antiphospholipid syndrome presenting as co-existing bilateral extensive pulmonary embolism and intracardiac mass with recurrence of thromboembolic phenomenon.

## Case presentation

A 23-year-old male patient, a medical student with no previously known co-morbids, presented to emergency room with bilateral chest pain, massive hemoptysis and cough. Vitals at initial assessment were: blood pressure (BP) 130/70 mmHg, pulse 85 beats per minute, temperature 98.6°F, respiratory rate (RR) 18/minute and SpO_2_ 97% at room air. On examination, 15/15 on Glasgow coma scale (GCS), auscultation of lungs revealed decreased breath sounds bilaterally and cardiovascular exam was normal. Electrocardiogram (ECG) was unremarkable except sinus tachycardia. There was no lymphadenopathy or hepatosplenomegaly. Initial investigations showed mild thrombocytopenia 113,000/mL. Coagulation profile including prothrombin time (PT) with international normalized ratio (INR), partial thromboplastin time (PTT), and fibrinogen were within normal range. Hepatitis profile was negative and chest X-ray showed wedge-shaped consolidations.

He had been having exertional dyspnea, right-sided chest pain which aggravated on inspiration and cough for about one month. He developed hemoptysis one week ago. Computed tomography (CT) scan done in another health facility was reported as having multiple peripheral pleural-based consolidation in the apical segment of right upper lobe, lateral basal segment of right lower lobe, lateral segment of right middle lobe, and apical segment of left lower lobe with surrounding halo representing pulmonary hemorrhage. He was being treated initially as pneumonia with antibiotics and pain killers which resolved his symptoms temporarily except exertional dyspnea, three days prior to the presenting episode. He had no history of weight loss, no allergies, no family history of bleeding disorders and no history of illicit drug use.

**Figure 1:**
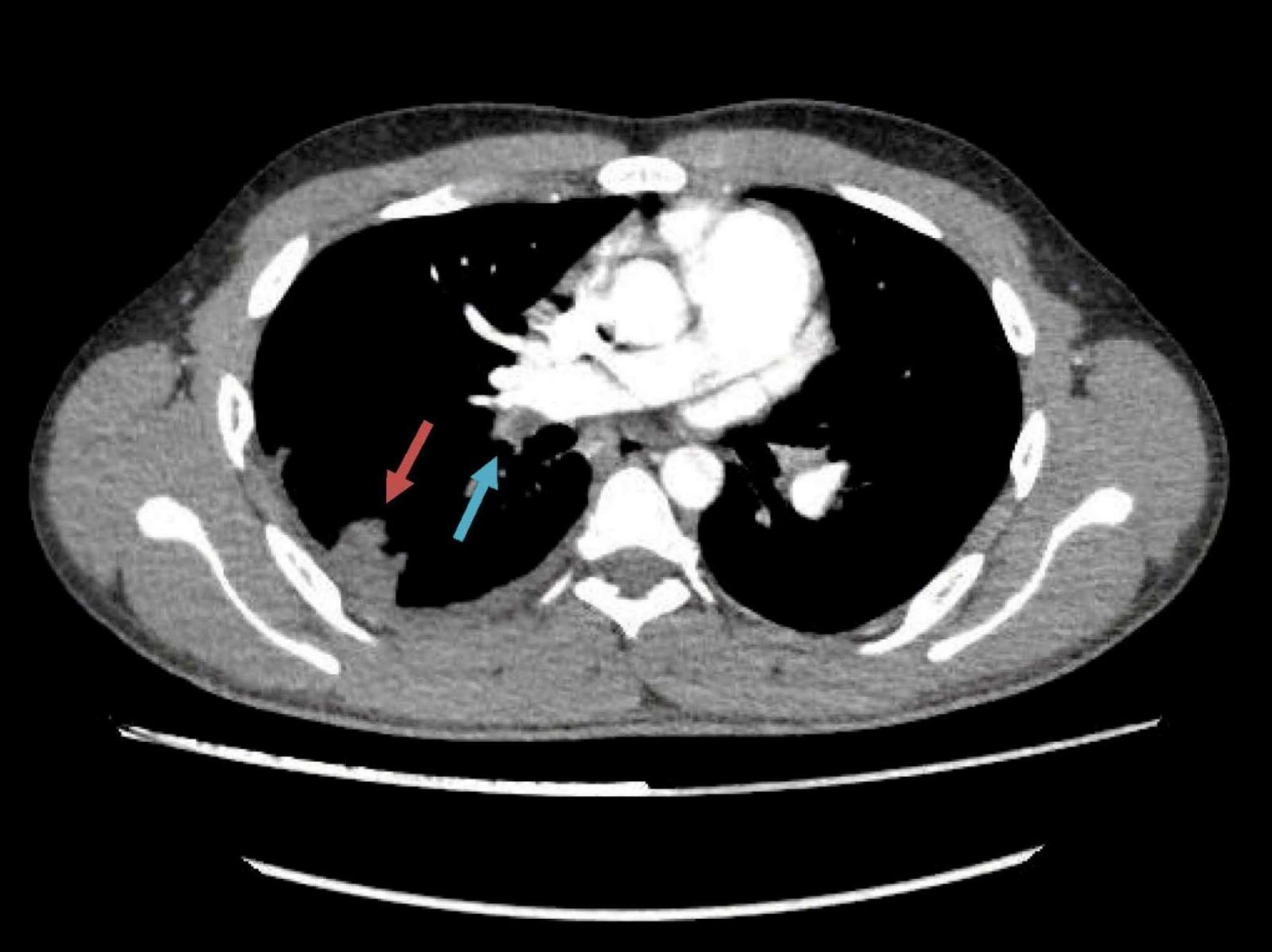
Contrast-enhanced computed tomography pulmonary angiogram. Blue arrow shows a filling defect in distal most right main pulmonary artery suggestive of pulmonary embolism. Red arrow shows wedge shape area of dense consolidation in right lateral basal segment representing infarct.

**Figure 2:**
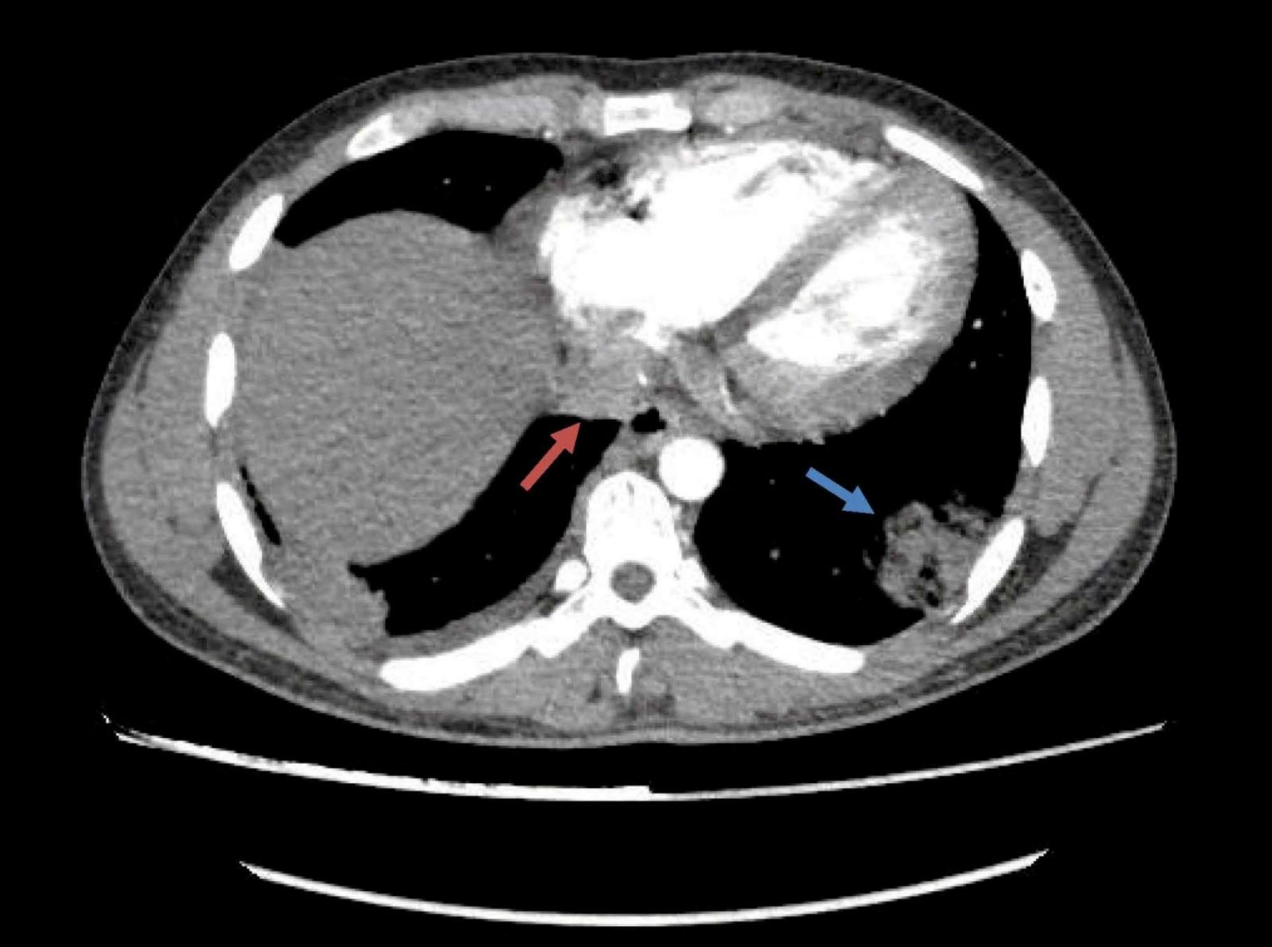
Contrast-enhanced computed tomography pulmonary angiogram. Red arrow shows a large filling defect in right atrium. Blue arrow shows dense consolidation in left lateral basal segment suggestive of infarct.

**Figure 3:**
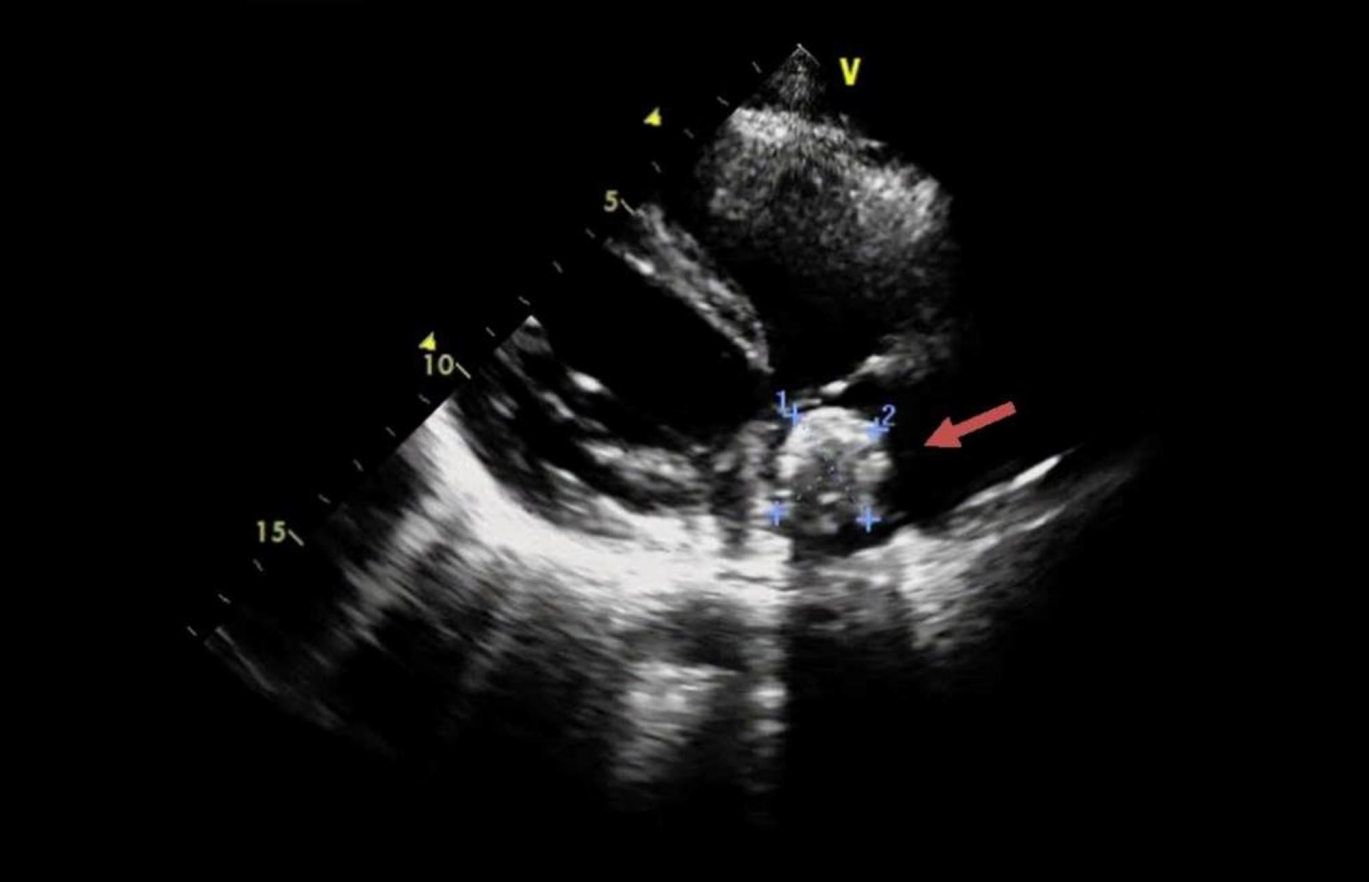
Two-dimensional transthoracic echocardiogram. Red arrow shows a well-defined 28 × 28 mm heterogeneous consistency attached to right side of the right atrium in fossa ovalis.

Workup upon admission revealed lupus coagulant to be strong positive, LA1/LA2 ratio to be 2.6 (less than 1.4), anticardiolipin antibodies IgG > 280 GPL/mL (>80 strong positive) and IgM 4.8 MPL/mL (>80 strong positive). Antinuclear antibody and extractable nuclear antibody (ENA) profiles were negative. Antithrombin III, protein C and protein S, liver function tests were within normal limits. CT pulmonary angiogram showed extensive bilateral pulmonary embolism with resultant lung infarcts more pronounced on right side (Figure 1), and a large filling defect in right atrium adjacent to posterior wall and closely abutting right atrioventricular valve (Figure 2). Deep venous thrombosis was ruled out by CT venogram of lower extremity from pelvis up to the level of knees. Transthoracic echocardiography followed by transesophageal echo showed a large rounded mass of 28 × 28 mm of heterogeneous consistency attached to right side of the right atrium in fosse ovalis area with dilatation of right atrial chamber. Rest of the echo was normal. It was suspected to be atrial myxoma (Figure 3). Normal procalcitonin level 0.09 ng/mL (less than 0.1 ng/mL) and sterile blood and urine cultures made infection less likely. CT scan abdomen and pelvis was done to rule out any metastatic process.

Treatment with therapeutic dose of anticoagulation was initiated. The patient was shifted to critical area and electively intubated following massive hemoptysis and respiratory compromise. Pulmonary angiography and subsequent embolization of right bronchial artery was performed. Excision of intracardiac lesion was done. Intraoperatively, right atrial lesion and fibrous tissue present on the posterior aspect of inferior vena cava orifice were resected and sent for histopathology. Anticoagulation with warfarin was resumed postoperatively with the target INR of 1.5 to 2. The right atrial mass on histopathology was consistent with embolus showing fibrinous tissue with dystrophic calcification. Postoperative extubation was uneventful and the patient was discharged.

**Figure 4:**
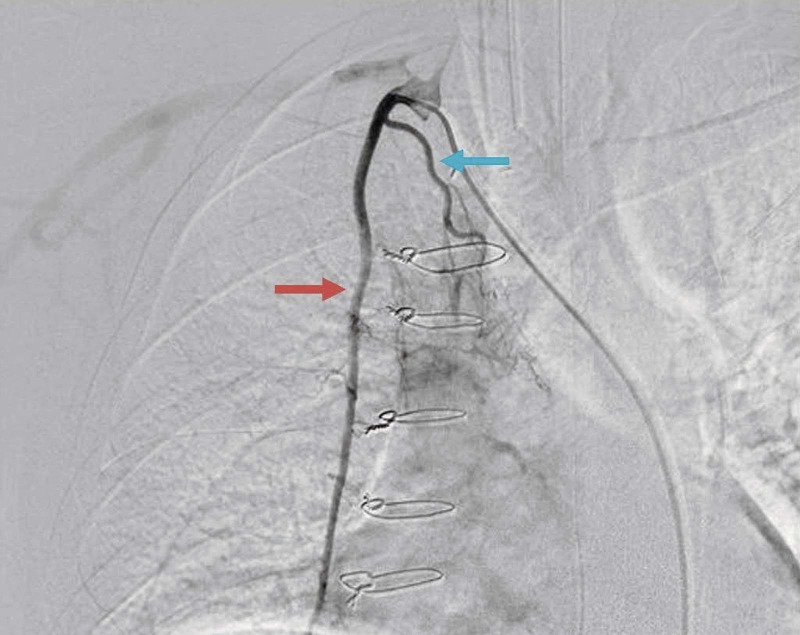
Bronchial artery angiography through selective catheterization of right subclavian artery. Red arrow shows contrast enhanced internal thoracic artery. Blue arrow shows anomalous origin of right bronchial artery from internal thoracic artery.

The patient presented to emergency room with massive hemoptysis (500 ml) 24 hours after discharge, again intubated due to hypoxia and for airway maintenance. His blood count was within normal range, and INR 2.7. Anticardiolipin antibody was >280 whereas lupus anticoagulant was not significant. Follow-up echocardiography ruled out recurrence of intracardiac thrombus. Pulmonary angiography showed abnormal origin of right bronchial artery from internal thoracic artery with abnormal vascularity and parenchymal blush (Figure 4) followed by embolization on the right bronchial artery in the right upper lobe. Considering near catastrophic antiphospholipid syndrome (CAPS), the patient was treated with pulse methylprednisolone, seven sessions of plasma exchange were done with three liters of fresh frozen plasma. Anticoagulation was withheld due to ongoing hemoptysis. Once bleeding stopped and the patient was extubated, intravenous heparin was initiated followed by maintenance with oral vitamin K antagonist. As the patient was hemodynamically stable with no further bleeding, he was discharged with regular follow-up in anticoagulation clinic.

## Discussion

APLS, also called Hughes syndrome, is a multisystem autoimmune disorder of hypercoagulation [4]. It is regarded as primary APLS if there is no underlying connective tissue disorder and secondary APLS if there is a history of connective tissue disorder such as systemic lupus erythematosus. It occurs most commonly in females with a female to male ration being 3.5:1 [2]. About 9% patients with APLS develop pulmonary embolism. Antiphospholipid syndrome presenting as co-existing bilateral extensive pulmonary embolism and intracardiac mass mimicking atrial myxoma is rare [5] particularly in the absence of an underlying structural cardiac abnormality. In the present case, a young male patient with no personal history of any coagulation disorders, presents with massive bilateral pulmonary embolism and an intracardiac mass leading to a diagnosis of APLS. The diagnosis was made on the basis of clinical presentation, presence of antiphospholipid antibodies and histopathology of the intracardiac mass. Recurrent bleeding episodes and pulmonary emboli lead to a near catastrophic syndrome.

The pathogenesis of APLS has not been clearly defined. It is considered that antiphospholipid antibodies are mainly responsible for causing vascular damage in APLS. Antiphospholipid antibodies target vascular and plasma cell proteins. These antibodies interact with endothelial surfaces, clotting protein C, protein S, prothrombin, and activate platelets and complement cascade [6]. The mechanism which leads to the formation of an intracardiac mass in APLS is not clear due to paucity of evidence. Interaction of endocardial surface with circulating antiphospholipid antibodies is thought to play a role by disrupting the balance between thrombosis and fibrinolysis [7] and by inducing changes in endocardial surface factors. In most cases, there is an underlying hemostatic defect, abnormal blood flow or ventricular dysfunction, but in the present case there is no underlying cardiac pathology [3,8]. The development of thrombi and high risk of recurrent thrombotic events has been linked to high titers of lupus anticoagulant and anticardiolipin antibodies [9].

The diagnosis of APLS is two-dimensional based on laboratory criteria and clinical features. The laboratory criteria are based on the presence of one or more antiphospholipid antibodies, namely, lupus anticoagulant, anticardiolipin antibodies and antibodies against beta 2-glycoprotein on two or more separate occasions at least 12 weeks apart. The clinical features which will aid in diagnosis of APLS include arterial or venous thrombosis and recurrent pregnancy loss. Presence of one laboratory and one clinical feature is required to make a diagnosis of antiphospholipid syndrome. The criteria for diagnosis of CAPS including definite and probable CAPS are illustrated below (Table 1) [10].

**Table 1:** Classification criteria for catastrophic antiphospholipid syndrome. aPL: Antiphospholipid antibodies

Preliminary criteria for classification of catastrophic antiphospholipid syndrome (CAPS)
1) Evidence of involvement of three or more organs, systems and/or tissues
2) Development of manifestations simultaneously or in less than a week
3) Confirmation by histopathology of small vessel occlusion in at least one organ or tissue
4) Laboratory confirmation of the presence of antiphospholipid antibodies (lupus anticoagulant and/or anticardiolipin antibody)
Definite CAPS
All four criteria
Probable CAPS
1) All four criteria, except for only two organs, systems and/or tissue involvement
2) All four criteria, except for the absence of laboratory confirmation at least six weeks apart due to early death of a patient never tested for aPL before the catastrophic APS
3) 1, 2 and 4
4) 1, 3 and 4 and the development of a third event in more than a week but less than a month, despite anticoagulation

aPL positive patients who do not comply with criteria for definite and probable CAPS can be included in CAPS-like group. This group includes aPL antibodies positive patients with medium to large vessel thrombosis in two organs in the presence or absence of concurrent bleeding, microthrombosis with hemorrhage (can be pulmonary or adrenal), severe thrombocytopenia with or without bleeding, and severe HELLP syndrome and associated single organ thrombosis [11]. Treatment of intracardiac mass is surgical excision followed by oral anticoagulation with INR monitoring (Range 2-3) is recommended. The management of CAPS-like disease is more challenging and calls for input from rheumatologist, interventional radiologist, haematologist, nephrologist and critical care, plasma exchange and infectious disease experts. Combination of high dose steroids, plasmapheresis or intravenous immunoglobulins and anticoagulation was found to be effective in increasing survival [12].

## Conclusions

Simultaneous occurrence of pulmonary embolism and intracardiac mass in antiphospholipid syndrome is a rare presentation. It is a treatable condition provided prompt diagnosis and early treatment is commenced. It is important to consider CAPS and CAPS-like conditions in young patients with antiphospholipid syndrome presenting with prothrombotic conditions such as pulmonary embolism, an intracardiac mass particularly in the absence of underlying cardiovascular diseases or risk factors. In addition, it is important to anticipate CAPS-like disease in patients with multiorgan involvement and known triggering factors like infections or postoperative period, in order to halt mortality by early diagnosis and aggressive treatment.
